# Clinical effects of two ozone therapy administration methods following mandibular third molar extraction: a parallel-group randomized clinical trial with exploratory salivary biomarker assessment

**DOI:** 10.1007/s00784-026-06834-7

**Published:** 2026-03-23

**Authors:** Monique Gonçalves da Costa, Eduardo Dallazen, Mateus Diego Pavelski, Izabela Fornazari Delamura, Larissa Victorino Sampaio, Antonio Hernandes Chaves-Neto, Leonardo Perez Faverani

**Affiliations:** 1 Department of Diagnosis and Surgery, Aracatuba School of Dentistry, Sao Paulo State University (UNESP), Aracatuba, SP 16015-050 Brazil; 2 Department of Basic Sciences, School of Dentistry, Sao Paulo State University (UNESP), Aracatuba, SP Brazil; 3 Department of Oral Diagnosis, Piracicaba Dental School, Universidade Estadual de Campinas (UNICAMP), Piracicaba, 13414-903 Brazil

**Keywords:** Surgery oral, Ozone, Saliva, Molar third

## Abstract

**Introduction:**

Some non-drug methods have been used as adjuncts to minimize tissue damage after third molar extractions, including ozone therapy, although there is no consensus on the most efficient protocol.

**Objectives:**

to compare two ozone therapy administration protocols regarding clinical parameters (edema, pain, maximum mouth opening) and salivary biomarkers of tissue injury and redox state.

**Materials and methods:**

This randomized, parallel-group clinical trial with blinded outcome assessment included thirty-five volunteers (45 mandibular third molars; 15 teeth per group). Participants were allocated to OZO-GAS (0.1 mL of subperiosteal ozonized gas), OZO-OIL (600 mEq of topical ozonized oil), or PLACEBO, administered immediately postoperatively and after 2 days. Outcome evaluation was performed by an examiner blinded to group allocation. Clinical outcomes were recorded at 24 h, 48 h, and 7 days, and unstimulated saliva was collected at the same time for spectrophotometric analyses.

**Results:**

The OZO-OIL group showed greater efficacy in reducing postoperative pain compared with PLACEBO (*p* < 0.05; Holm–Sidak). At 48 h postoperatively, OZO-OIL was associated with reduced salivary protein carbonyls and TBARS, alongside increased uric acid levels, compared with the PLACEBO group (*p* < 0.05). No consistent clinical or biochemical benefits were observed for the OZO-GAS protocol, and salivary related enzymes (AST, ALT, and ALP) suggested a less favorable biochemical profile in this group .

**Conclusions:**

Topical ozonized oil improved postoperative pain following mandibular third molar extraction and was associated with trends toward reduced oxidative damage and enhanced antioxidant defense compared with placebo, while gas administration showed no comparable benefits. The observed biochemical effects should be considered exploratory and correlated with the clinical findings.

**Clinical relevance:**

Ozonized oil represents a simple, non-pharmacological adjuvant capable of enhancing postoperative recovery after third molar extraction.

## Introduction

Surgical extraction of impacted third molars is one of the most commonly performed procedures by oral and maxillofacial surgeons in clinical practice [1–3]. This procedure involves trauma to the soft and hard tissues and may result in considerable pain, edema, and trismus during the postoperative period [4–6]. The literature has shown that the impact of third molar extraction on quality of life can be up to three times greater in patients who experience painful symptoms, edema, and trismus [7] (alone or in combination) compared with those who remain asymptomatic postoperatively [3, 8, 9].

Such evidence highlights the need to develop new studies on protocols that assist in controlling pain, edema, and trismus in patients undergoing third molar surgery [7, 10, 11]. Some non-pharmacological methods have been used as adjunctive therapies in an attempt to minimize tissue damage after third molar extraction, including cryotherapy, low-level laser therapy, and ozone therapy [9, 10, 12].

Ozone (O₃) has antimicrobial, immunostimulatory, antihypoxic, and biosynthetic effects. It can be administered through different routes, including gas, water, and oil [13–16]. In its gaseous form, O₃ is generated by devices using either an open system or a sealed suction system. In this form, it is administered via insufflation, which may be rectal or applied to the nasal, tubal, oral, vaginal, vesical, pleural, and peritoneal cavities [16, 17]. Ozonized oil may be advantageous and provide greater tissue permeation [13]. When combined with ozonized water treatment in clinical settings, it significantly improves the outcomes of O₃ therapy in controlling bacteria, viruses, and cutaneous fungal infections [18].

Although ozone therapy presents well-documented beneficial effects on wound healing and inflammatory control, there remains a lack of studies directly comparing different ozone administration modalities in the context of oral surgery [16, 19]. In particular, gaseous ozone and ozonized oil are widely used; however, their differences in terms of local bioavailability, residence time at the surgical site, and impact on the postoperative inflammatory response remain poorly understood. Most available studies evaluate these modalities in isolation, limiting the ability to determine which presentation provides superior clinical outcomes following third molar extraction [20–22].

Surgical wound repair is a complex process involving growth factors, enzymes, cytokines, and chemokines that accumulate in the surgical site during the inflammatory phase and regulate cell migration and the cellular infiltrate required for tissue repair [23–25]. In this context, saliva emerges as an auxiliary tool for prevention and diagnosis, notable for its ease of collection, favorable cost–benefit ratio, and wide availability. It is also highly useful for postoperative follow-up and for implementing adjunctive therapies after oral surgery [26–28].

The selection of salivary biomarkers was based on pathophysiological hypotheses related to surgical stress, tissue inflammation, and oxidative stress triggered by third molar extraction, as well as on the potential modulatory effects of ozone therapy on these processes. It was hypothesized that surgical trauma and the acute inflammatory response promote autonomic activation, local cellular damage, and redox imbalance, which may be reflected by measurable changes in salivary composition.

Accordingly, salivary total protein (TP) was included based on the hypothesis that the physical and psychological stress associated with the surgical procedure, as well as acute post-extraction inflammation, induces increased autonomic nervous system activity, resulting in elevated salivary TP levels [29–31]. Additionally, it was considered that interventions capable of modulating the local inflammatory response could attenuate these alterations.

The enzymes total alkaline phosphatase (TAP), alkaline phosphatase (ALP), aspartate aminotransferase (AST), and alanine aminotransferase (ALT) were selected under the hypothesis that surgical trauma promotes cellular damage and inflammation in oral tissues, leading to increased activity of these enzymes in saliva. These markers have been associated with alveolar bone destruction and tissue degradation in inflammatory conditions such as advanced periodontal disease and may reflect similar alterations during the postoperative period following tooth extraction [32, 33].

Considering that saliva constitutes an important first line of defense against oxidative stress, biomarkers related to redox balance were included based on the hypothesis that tooth extraction triggers increased local and systemic oxidative stress [24, 34, 35]. Uric acid (UA), the main non-enzymatic antioxidant in saliva and responsible for approximately 70% of total antioxidant capacity (TAC), was selected together with salivary TAC to assess the antioxidant response to the surgical insult [24, 28]. In addition, thiobarbituric acid reactive substances (TBARS) were used under the hypothesis that surgery-induced oxidative damage would result in increased lipid peroxidation detectable in saliva [33, 36]. Although previous studies have evaluated salivary inflammatory markers after tooth extraction, the potential modulatory effect of ozone therapy on salivary redox balance and enzymatic markers of tissue injury remains poorly understood. Thus, we hypothesized that ozone therapy modulates the acute inflammatory and oxidative response induced by third molar extraction, leading to measurable reductions in salivary markers of autonomic activation, tissue damage, and lipid peroxidation, along with preservation of antioxidant capacity.

Thus, covering the context of Oral and Maxillofacial Surgery and Traumatology, this proposal finds an important investigative justification in its potential to add knowledge to the clinic of adjuvant therapeutic strategies for controlling symptoms following third molar extraction which do not induce local and systemic side effects.

## Proposition

As a general objective, we proposed to compare the efficiency of two ozone therapy administration protocols—subperiosteal insufflation and ozonized oil—in controlling symptoms after removal of third molars.

Specific objectives included:

1. To clinically compare the efficacy of the protocols in controlling postoperative symptoms (pain, edema, and maximum mouth opening), defined as *primary outcomes*;
2. To analyze the concentration of salivary biomarkers indicating tissue injury (TP, ACP, ALP, AST and ALT) and oxidative stress (PC, TBARS, TAC and UA) present in the saliva of patients in each experimental group at each time of analysis, defined as *secondary outcomes*.

As for null hypothesis, we posited that the test groups (subperiosteal insufflation and ozonized oil) would present no differences in relation to the control group (placebo) for the clinical and salivary biochemical parameters analyzed.

## Materials and methods

### Research design

The research project consisted of a randomized, simple-blind, parallel-group clinical study. It was submitted to the Research Ethics Committee, CAAE 60799022.1.0000.5420, and to the Brazilian Registry of Clinical Trials, ReBEC No. RBR-8zypz4p. All 35 volunteers of both genders, received information about the study, its objectives and possible risks, and signed an informed consent form prepared based on all the items described in the National Health Council Guidelines (CNS Resolution 196/96).

Blinding was applied only to the evaluators responsible for outcome collection and analysis, and participant blinding was not feasible due to the nature of the intervention. Additionally, no sham intervention was employed, which may represent a methodological limitation.

### Sample selection

A total of 35 volunteers of both genders and with unilateral or bilateral mandibular third molars were selected to participate in the study. All surgical procedures and salivary sample collection were performed at the surgical clinics of the Dental Care Center for Patients with Special Needs (CAOE), School of Dentistry of Aracatuba – UNESP.

Due to the absence of previous clinical trials employing a comparable methodology and similar salivary biochemical outcomes, a formal a priori sample size calculation based on a clearly defined primary endpoint was not performed. Therefore, this study should be considered exploratory in nature. The sample size was determined based on previously published studies with analogous experimental designs and outcome measures, which reported homogeneous results using approximately 15 samples (teeth) per group [1, 37]. Although this strategy is commonly adopted in pilot and exploratory clinical investigations when effect size estimates are unavailable, the lack of a predefined power calculation must be acknowledged when interpreting the findings.

### Inclusion and exclusion criteria

Inclusion criteria for patient selection, based on anamnesis, clinical and radiographic examinations [38–41], consisted of:

- Patients in adequate systemic and local health conditions to undergo the proposed surgical procedures;
- Age between 16 and 35 years;
- Indication for extraction of mandibular third molars (teeth 38 and/or 48), classified as position A or B and class I or II according to Pell and Gregory, with at least two-thirds of root formation [42, 43].

Exclusion criteria, established through anamnesis and clinical and radiographic examination [38, 44–46], included:

- Presence of local pathological conditions such as pericoronitis, cysts, odontogenic tumors, trauma, active infection, or periodontal disease associated or not with the third molar;
- Smoking, alcohol consumption, or other substance abuse;
- History of hypersensitivity to any medication used in the study;
- Women who were menstruating, pregnant, or breastfeeding during the study period;
- Use of psychiatric medications, corticosteroids, estrogens, or androgens.

Randomization was performed at the tooth level. In patients requiring bilateral mandibular third molar extractions, each tooth was considered an independent experimental unit and was randomly allocated to one of the study groups. For patients undergoing unilateral extraction, the single tooth was randomized accordingly.

Allocation concealment was ensured using a computer-generated randomization sequence prepared by an independent researcher not involved in the surgical procedures or outcome assessment. Group assignments were placed in sequentially numbered, opaque, sealed envelopes, which were opened only at the time of intervention.

### Test groups

The selected individuals were randomized into three groups of 15 people (*n* = 15 teeth) according to the established therapeutic protocol. The groups were identified as: OZO-GAS (administration of 0.1 ml of ozonized gas via subperiosteal route); OZO-OIL (administration of 600 mEq of ozonized oil via topical route) and PLACEBO (no therapy).

Tooth extractions were standardized, consisting of a triangular incision, osteotomy, and, when necessary, odontosection. All surgeries were performed by the same surgeon and assistant.

### Group randomization and blinding

Randomization was performed using sealed envelopes separated by groups and conducted by a participant external to the surgery. Allocation was made according to the therapy applied in the postoperative period (OZO-GAS, OZO-OIL, or PLACEBO). In cases where the patient had both lower third molars, the initial side of the surgery was also defined by randomization, using an envelope containing two pieces of paper labeled “right side” and “left side.”

The volunteers were not blinded, as they were able to identify the therapy received either by the characteristic taste of the oil, by the sensation of puncturing and insufflation in the case of gas, or by the absence of intervention in the placebo group. However, both the surgeon responsible for the procedure and the researcher in charge of clinical, biochemical, and statistical analyses were unaware of the distribution of the experimental groups, characterizing the study as sample-blind.

### Surgical procedure

Extraoral antisepsis was performed with 0.5% alcoholic chlorhexidine and intraoral antisepsis with 0.12% chlorhexidine digluconate by rinsing for 60 s. Subsequently, regional anesthesia was administered to the inferior alveolar, buccal, and lingual nerves using 2% mepivacaine combined with epinephrine 1:100,000 (Mepiadre^®^, DFL – Brasil). A triangular mucoperiosteal incision was made with a No. 15 blade, with relaxation mesial to the lower second molar. Osteotomy was performed with a No. 702 drill at high speed, under continuous irrigation with sterile 0.9% NaCl.

Extraction was performed using curved and straight extractors, followed by inspection of the socket with a Lucas curette to remove the follicle and bone spicules. When necessary, bone regularization was performed with a file, finishing with irrigation and suturing with 5.0 nylon (Johnson & Johnson, Brasil). All surgeries were performed between 8 and 10 a.m. to minimize circadian rhythm variations. Patients who had all four third molars were scheduled for unilateral surgery with a minimum interval of 21 days between procedures.

After surgery, patients were instructed on post-surgical care and prescribed standard antibiotic therapy (amoxicillin, 500 mg every 8 h for 7 days, p.o.) and for patients who were allergic to penicillin, clindamycin, 300 mg every 8 h for 7 days, p.o., was prescribed, in addition to sodium dipyrone (500 mg, p.o.), for 3 days in case of pain. Cases in which there were any type of post-surgical complications, patients were treated and then excluded from the research analyses.

### Ozone therapy

Immediately after tooth extraction, patients allocated to the OZO-GAS and OZO-OIL groups received ozone therapy. The dosage, volume, and timing of ozone application were defined based on previous clinical and experimental studies demonstrating biological activity within a therapeutic window that minimizes cytotoxic effects while promoting antimicrobial, anti-inflammatory, and tissue repair responses [19, 44].

In the OZO-GAS group, ozone (O₃) was applied by subperiosteal insufflation using an ozone generator (OZONE&LIFE INDÚSTRIA, COMÉRCIO E SISTEMAS LTDA, São José dos Campos, SP, Brazil). A volume of 0.1 mL was administered at a distance of 1 cm from the surgical site using a 30 mL disposable syringe and a 25 mm × 0.7 mm needle, immediately after surgery and on the second postoperative (PO) day. This protocol was selected to ensure local delivery of ozone at low volume, reducing the risk of tissue irritation while maintaining biological efficacy.

In the OZO-OIL group, ozonized sunflower oil (30 mL, 600 meq) (PHILOZON INDÚSTRIA E COMÉRCIO DE GERADORES DE OZÔNIO, Balneário Camboriú, SC, Brazil) was applied topically. A volume of 0.5 mL was distributed throughout the alveolar socket using a 1 mL syringe and a 13 mm × 0.38 mm needle, covering the entire alveolar surface for 2 min. The chosen volume and exposure time were based on prior studies demonstrating effective antimicrobial and biostimulatory effects of ozonized oils in oral wound healing. Applications were performed immediately after surgery and on the second PO day.

Although the routes of administration differ in invasiveness, both protocols aimed to deliver ozone locally during the early inflammatory phase of wound healing, a period characterized by high microbial challenge and oxidative signaling relevant to tissue repair. The second PO application was intended to reinforce these biological effects during the transition to the proliferative phase. Patients allocated to the PLACEBO group did not receive any adjunctive therapy during the experimental period.

### Postoperative clinical parameters

A single examiner, previously trained and blinded to group allocation, performed all clinical evaluations. Edema, trismus, and postoperative pain were assessed preoperatively and at 2 and 7 days after surgery. Facial edema was measured using a flexible ruler along two reference axes: from the corner of the mouth to the earlobe and from the outer canthus of the eye to the mandibular angle [47–49]. Trismus was quantified by calculating the variation in maximum mouth opening between the preoperative and postoperative periods.

Pain intensity was assessed using a 10-cm visual analog scale (VAS), anchored by “no pain” and “worst imaginable pain.” Analgesic consumption was standardized by dispensing medication in individualized packaging. Participants recorded daily intake, and unused tablets were returned at the 7-day postoperative visit for compliance verification.

### Saliva collection and processing

Saliva collection followed the protocol adapted from Ozmeric et al. [25], on the same days as the clinical evaluations (preoperative, 2 and 7 days post-surgery). Unstimulated whole saliva samples were collected between 8 a.m. and 9 a.m. to minimize circadian variations. Participants brushed their teeth without toothpaste and fasted for 2 h prior to collection. After rinsing the mouth with water, saliva was expectorated every 60 s into sterile tubes until 5–8 mL was collected, discarding the first minute. The samples were then centrifuged at 3000 rpm for 10 min at 4 °C (Eppendorf R 5810, Hamburg, Germany), and the resulting supernatant was fractionated into aliquots and stored at − 80 °C until biochemical analysis.

### Biochemical analysis of saliva

All reagents used for the biochemical analyses were obtained from Sigma-Aldrich (Germany/USA) and Bioclin (Belo Horizonte, MG, Brazil). Absorbance readings were performed using a UV–Vis spectrophotometer (UV-1203, Shimadzu, Japan) and a microplate reader (PowerWave 340, BioTek, USA), following proper calibration procedures.

To minimize inter-assay variability, all samples were processed on the same day under identical experimental conditions. Whenever applicable, commercial kits from the same manufacturing lot were used to reduce potential batch-related variation. All measurements were performed in technical duplicates, and the mean of the two readings was used for statistical analysis, ensuring intra-assay reliability. Biochemical results were normalized to total protein content to allow proportional comparisons among samples [50].

### TP concentration

TP concentration was determined by the Lowry method, using bovine serum albumin as a standard, as described by Hartree [51, 52]. Results were expressed as g/L. All analyses performed were normalized by total protein content.

### Phosphatase activity (ACP and ALP)

Total acid phosphatase (ACP) activity was determined by the hydrolysis of p-nitrophenyl phosphate (*p*-NPP) to p-nitrophenol (*p*-NP) at pH 5.0, whereas alkaline phosphatase (ALP) activity was measured at pH 9.4 in the presence of magnesium chloride [53, 54]. *p*-NP formation was quantified spectrophotometrically at 405 nm, using a molar extinction coefficient of 18,000. M⁻¹.cm⁻¹. Enzyme-free control tests were performed to correct for non-enzymatic hydrolysis. One unit of enzymatic activity was defined as the amount of enzyme capable of hydrolyzing 1 µmol of *p*-NPP per minute at 37 °C. Results were expressed as specific activity (U/g of total protein).

### AST and ALT activity

Salivary activity of the AST and ALT enzymes were determined using commercial kits (Bioclin, Belo Horizonte, Minas Gerais, Brazil) with a BIO-2000 biochemical analyzer (Bioplus, Barueri, São Paulo, Brasil). Values were expressed as U/g of total protein [55, 56].

### Salivary oxidative damage

PC concentration was quantified using the alkaline 2,4-dinitrophenylhydrazine (DNPH) method [57]. Carbonyl content was calculated using the molar extinction coefficient (ε450 = 22,308 M^− 1^ cm^− 1^). Results were expressed as µmol/g of total protein. Lipid peroxidation was assessed by TBARS concentration [58]. The amount of aldehydes formed was calculated by the molar extinction coefficient (ε532 = 1.56 × 10^5^ M^− 1^ cm^− 1^). Results were expressed as µmol/g of total protein.

### Total antioxidant capacity defense

TAC was determined using the iron reduction method, as previously described by Benzie and Strain [59]. Results were expressed as µmol of Fe^+ 2^/g total protein. UA concentration was measured using a commercial kit (Bioclin, Belo Horizonte, MG, Brasil), in which uricase was used in the assay to convert uric acid into allantoin and hydrogen peroxide. Results were expressed in mg/g of total protein [60].

### Statistical analysis

Statistical analyses were performed using SigmaPlot 12.0 (Systat Software Inc., San Jose, CA, USA). Data were expressed as mean ± standard error of the mean (SEM). The experimental design included two independent factors: group (PLACEBO, OZO-GAS, and OZO-OIL) and time (baseline, 2 days, and 7 days postoperatively), with repeated measurements over time for the same experimental unit. Therefore, a two-way analysis of variance (ANOVA) for repeated measures was applied to evaluate the main effects of group and time, as well as their interaction.

Normality of data distribution was assessed using the Shapiro–Wilk test. Homogeneity of variances was evaluated using Levene’s test, and the assumption of sphericity was verified using Mauchly’s test. When violations of sphericity were detected, the Greenhouse–Geisser correction was applied. Post hoc comparisons between groups and time points were performed using the Holm–Sidak test. A significance level of 5% (*p* < 0.05) was adopted for all analyses.

Considering the number of biochemical endpoints evaluated, the possibility of type I error inflation due to multiple comparisons is acknowledged. No additional formal correction for multiple testing across different endpoints was applied; therefore, these analyses should be interpreted as exploratory in nature.

## Results

### Clinical parameters and post-surgical evaluations

**Fig. 1:**
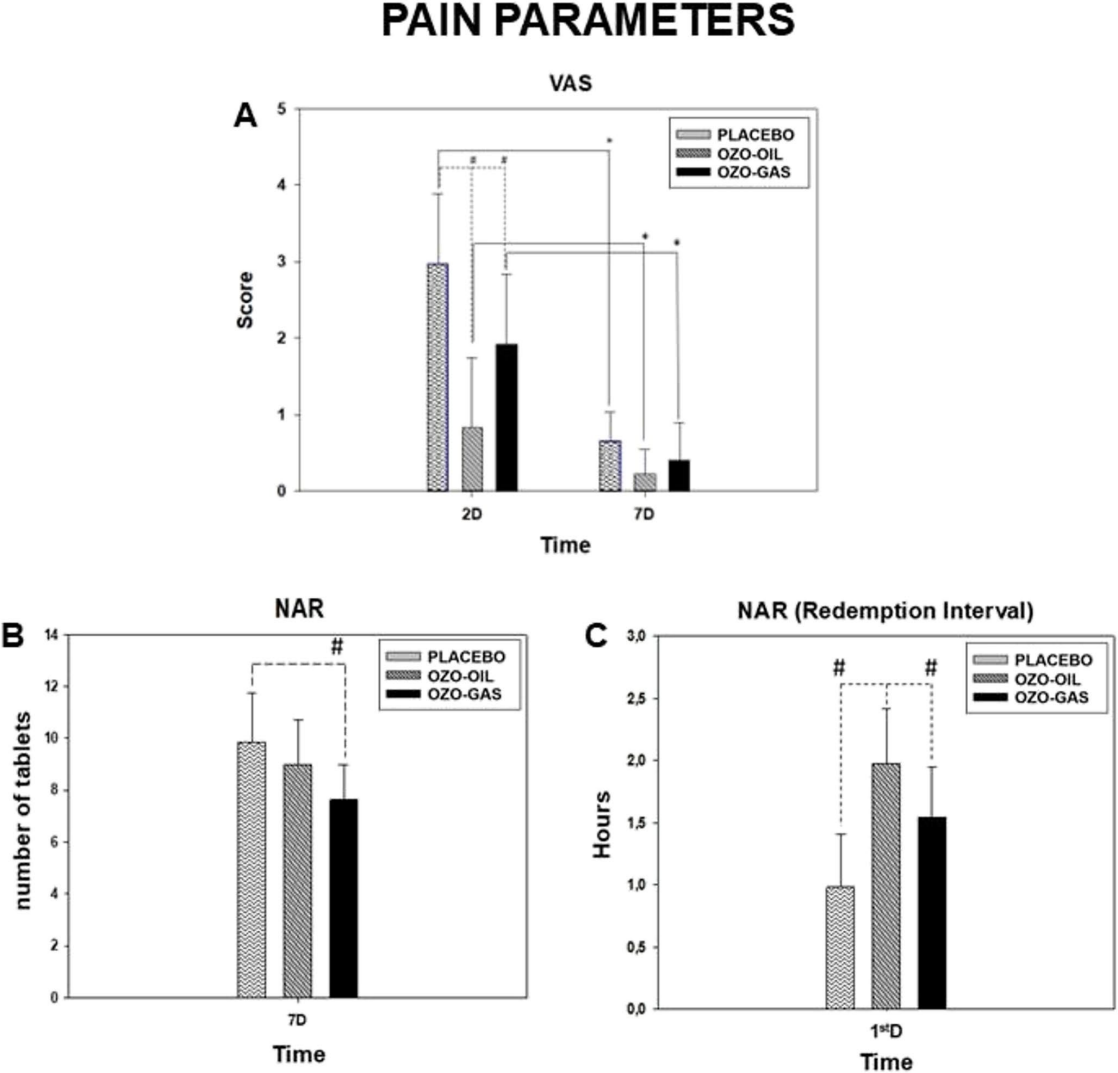
Representative graphs of the clinical parameters evaluated. Pain was assessed using the Visual Analog Scale (VAS), with data expressed as mean and standard error of the mean. The groups analyzed were PLACEBO, OZO-OIL, and OZO-GAS, evaluated at preoperative and postoperative (PO) periods. The symbol (*) indicates a statistical difference in the comparison between groups over time, while (#) denotes differences between groups within the same experimental period. NAR values are also presented for the same groups at **A** immediate PO and **B** 7th PO day, with (#) indicating differences between groups within each evaluated period

Pain assessment using VAS demonstrated significant postoperative changes in all groups. All participants reported no pain preoperatively, with pain peaking on postoperative day 2 and decreasing significantly by day 7. No intergroup differences were observed at baseline. On postoperative day 2, VAS scores differed significantly among groups, with higher pain levels in the PLACEBO group (2.97) compared with OZO-GAS (1.92) and OZO-OIL (0.92) (*P* < 0.05). By postoperative day 7, pain scores were low and comparable among PLACEBO (0.65), OZO-GAS (0.40), and OZO-OIL (0.22), with no statistically significant intergroup differences (Fig. 1).

NAR showed that the OZO-GAS group required fewer analgesic tablets (7.62) than PLACEBO (9.86) (*P* < 0.05), while OZO-OIL (8.97) did not differ significantly from either group. Regarding the time to first rescue analgesic, all groups differed significantly (*P* < 0.05), with the longest interval observed in OZO-OIL (1.97), followed by OZO-GAS (1.54) and PLACEBO (0.98) (Fig. 1).

**Fig. 2:**
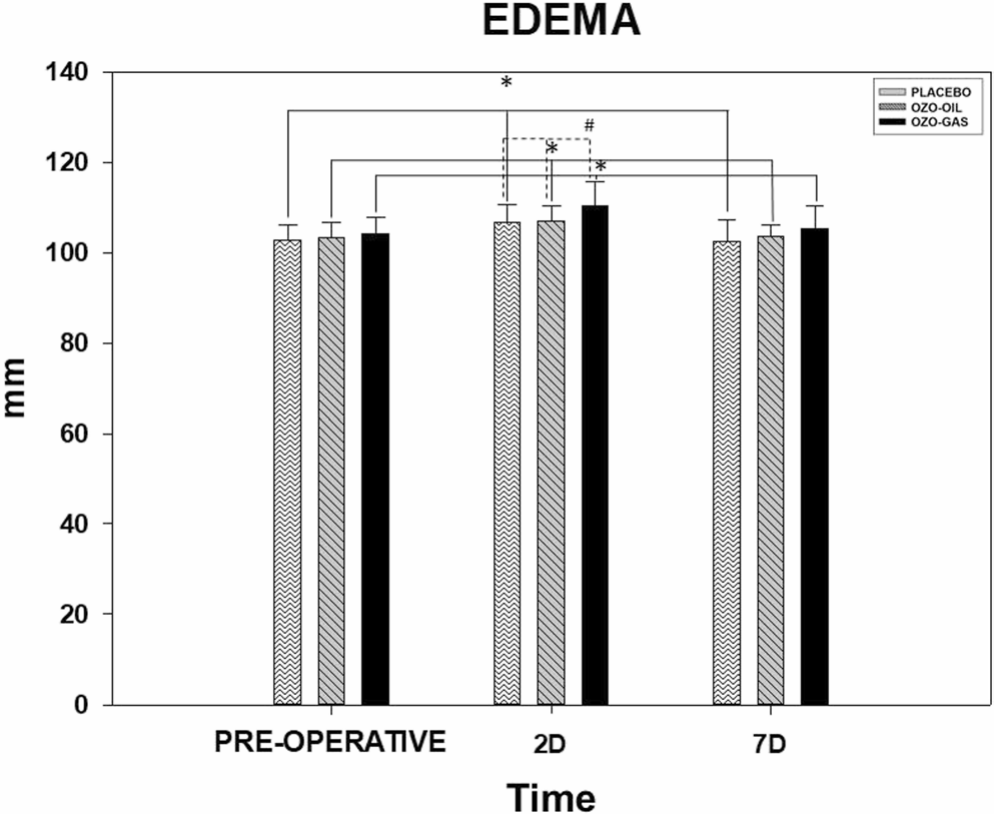
Representative graph of edema measurements, with data expressed as mean and standard error of the mean. The groups shown are PLACEBO, OZO-OIL, and OZO-GAS, evaluated in the preoperative and postoperative (PO) periods. The symbol (*) indicates a statistical difference in the group × time comparison, while (#) denotes differences between groups within the same experimental period

Edema assessment showed significant temporal changes in all groups (*P* < 0.05), with peak values observed on postoperative day 2 and a return to preoperative levels by day 7 (Fig. 2). No intergroup differences were observed at baseline. On postoperative day 2, the OZO-GAS group exhibited higher edema values (110.43) compared with PLACEBO (106.65) and OZO-OIL (107.00) (*P* < 0.05). By postoperative day 7, edema values were comparable among PLACEBO (102.44), OZO-OIL (103.58), and OZO-GAS (105.34), with no statistically significant intergroup differences.

**Fig. 3:**
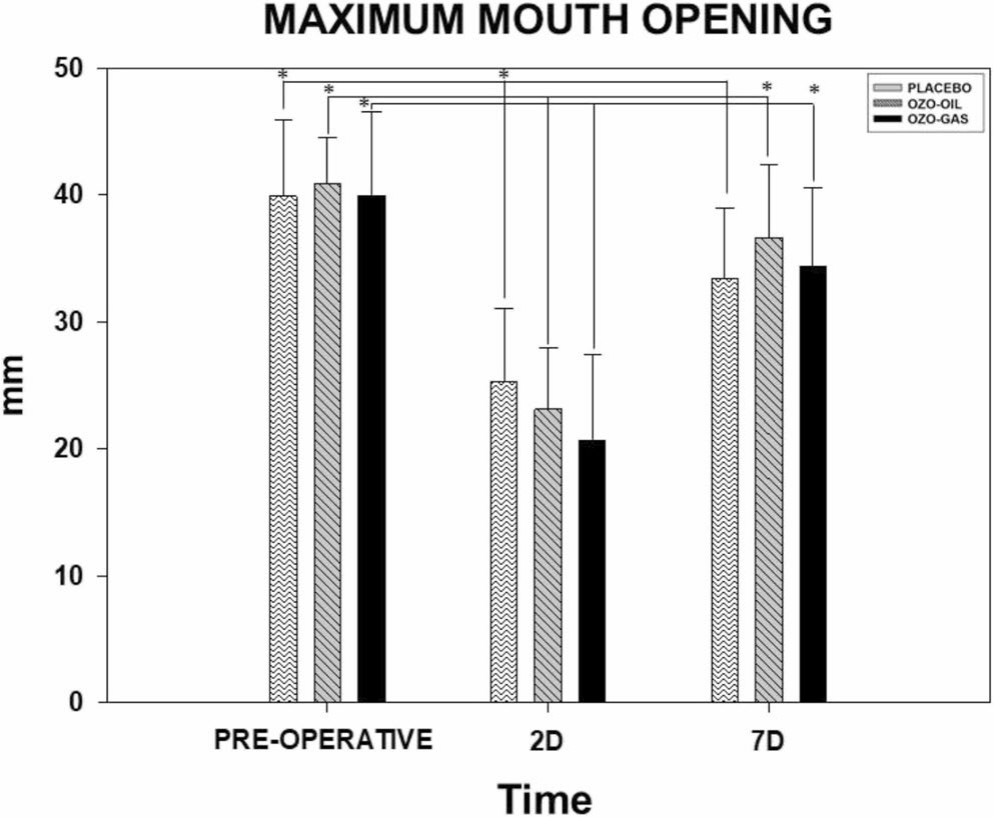
Representative graph of maximum mouth opening measurements, with data expressed as mean and standard error of the mean. The groups shown are PLACEBO, OZO-OIL, and OZO-GAS, evaluated in the preoperative and postoperative (PO) periods. The symbol (*) indicates a statistical difference in the group × time comparison

All groups exhibited a significant reduction in mouth opening during the postoperative period, with no statistically significant differences among PLACEBO, OZO-OIL, and OZO-GAS at any evaluated time point (*P* > 0.05). In the PLACEBO group, mean mouth opening decreased from 39.91 mm preoperatively to 25.26 mm on postoperative day 2 and partially recovered to 33.37 mm by day 7 (*P* < 0.05). Similarly, in the OZO-OIL group, mean mouth opening decreased from 40.91 mm at baseline to 23.12 mm on day 2, followed by recovery to 36.61 mm on day 7 (*P* < 0.05) (Fig. 3).

### Biochemical parameters

#### Effects of ozone therapy on salivary TP concentration after tooth extraction

**Fig. 4:**
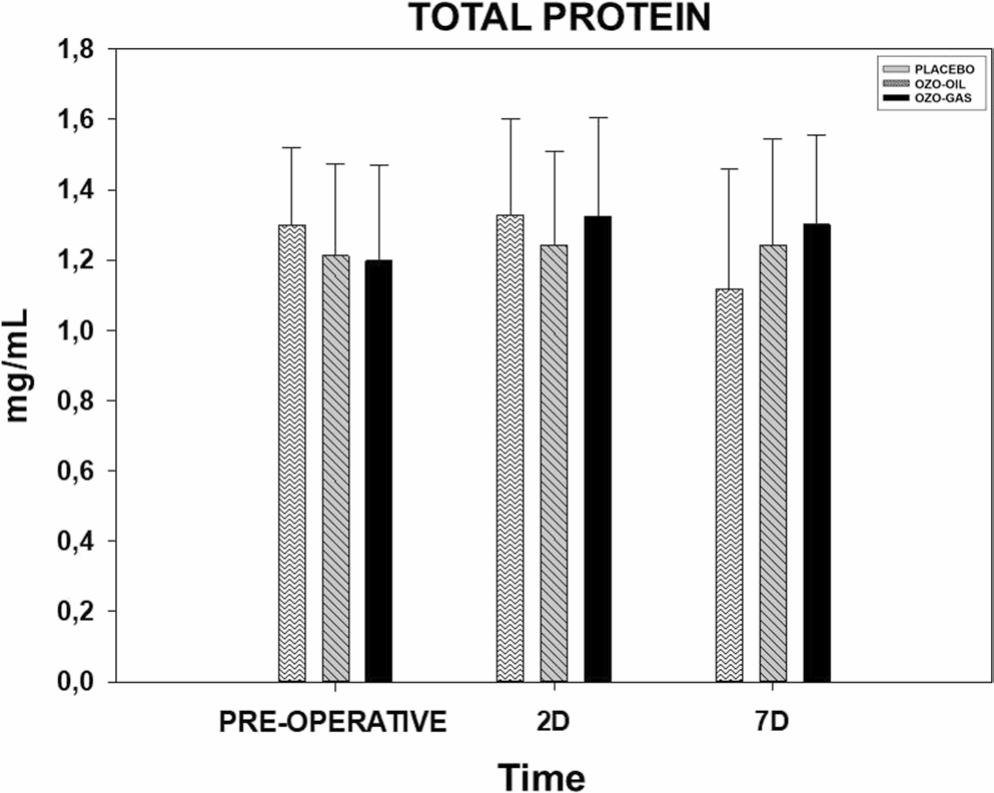
Total protein concentration, with data expressed as mean and standard error of the mean. The groups shown are PLACEBO, OZO-OIL, and OZO-GAS, evaluated in the preoperative and postoperative (PO) periods

Total protein concentrations did not show significant changes over time in any of the groups, with comparable values ​​observed preoperatively and on postoperative days 2 and 7. No statistically significant differences were detected between the groups; thus, PLACEBO = OZO-OIL = OZO-GAS (Fig. 4).

#### Effects of ozone therapy on salivary phosphatase activities after tooth extraction

**Fig. 5:**
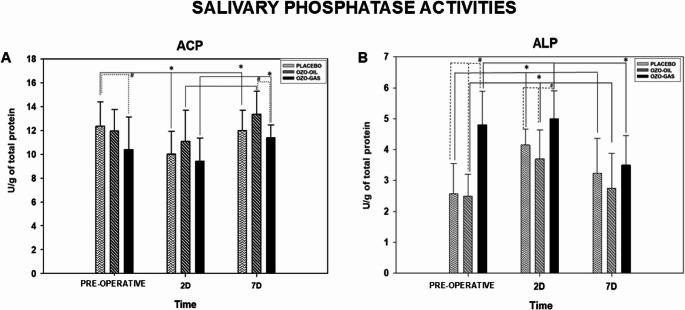
ACP activity and ALP activity for the PLACEBO, OZO-OIL, and OZO-GAS groups, evaluated in the preoperative and postoperative (PO) periods. The symbol (*) indicates a statistical difference in the group × time comparison, while (#) denotes differences between groups within the same experimental period

ACP activity showed significant temporal variation in all groups. In the PLACEBO group, ACP decreased on postoperative day 2 compared with baseline and returned to baseline levels by day 7 (*P* < 0.05). In the OZO-OIL and OZO-GAS groups, ACP also decreased on day 2 and increased by day 7, with significant differences between days 2 and 7 in both groups (*P* < 0.05). At baseline, ACP activity was higher in PLACEBO compared with both ozone groups (PLACEBO > OZO-GAS = OZO-OIL). No intergroup differences were observed on postoperative day 2. By day 7, ACP levels were higher in OZO-OIL than in OZO-GAS, while neither differed from PLACEBO (Fig. 5).

ALP activity showed significant temporal variations in all groups. In the PLACEBO group, ALP increased on postoperative day 2 compared with baseline and decreased by day 7, with a significant difference between days 2 and 7 (*P* < 0.05). In the OZO-OIL group, ALP also increased on day 2 and declined on day 7, with significant differences between day 2 and baseline as well as between days 2 and 7 (*P* < 0.05). In the OZO-GAS group, ALP levels were higher at baseline and on day 2, followed by a significant reduction on day 7 (*P* < 0.05). Intergroup analysis showed that OZO-GAS presented higher ALP activity than PLACEBO and OZO-OIL at baseline and on postoperative day 2 (OZO-GAS > PLACEBO = OZO-OIL). No intergroup differences were observed on postoperative day 7 (Fig. 5).

#### Effects of ozone therapy on salivary transaminase activities after tooth extraction

**Fig. 6:**
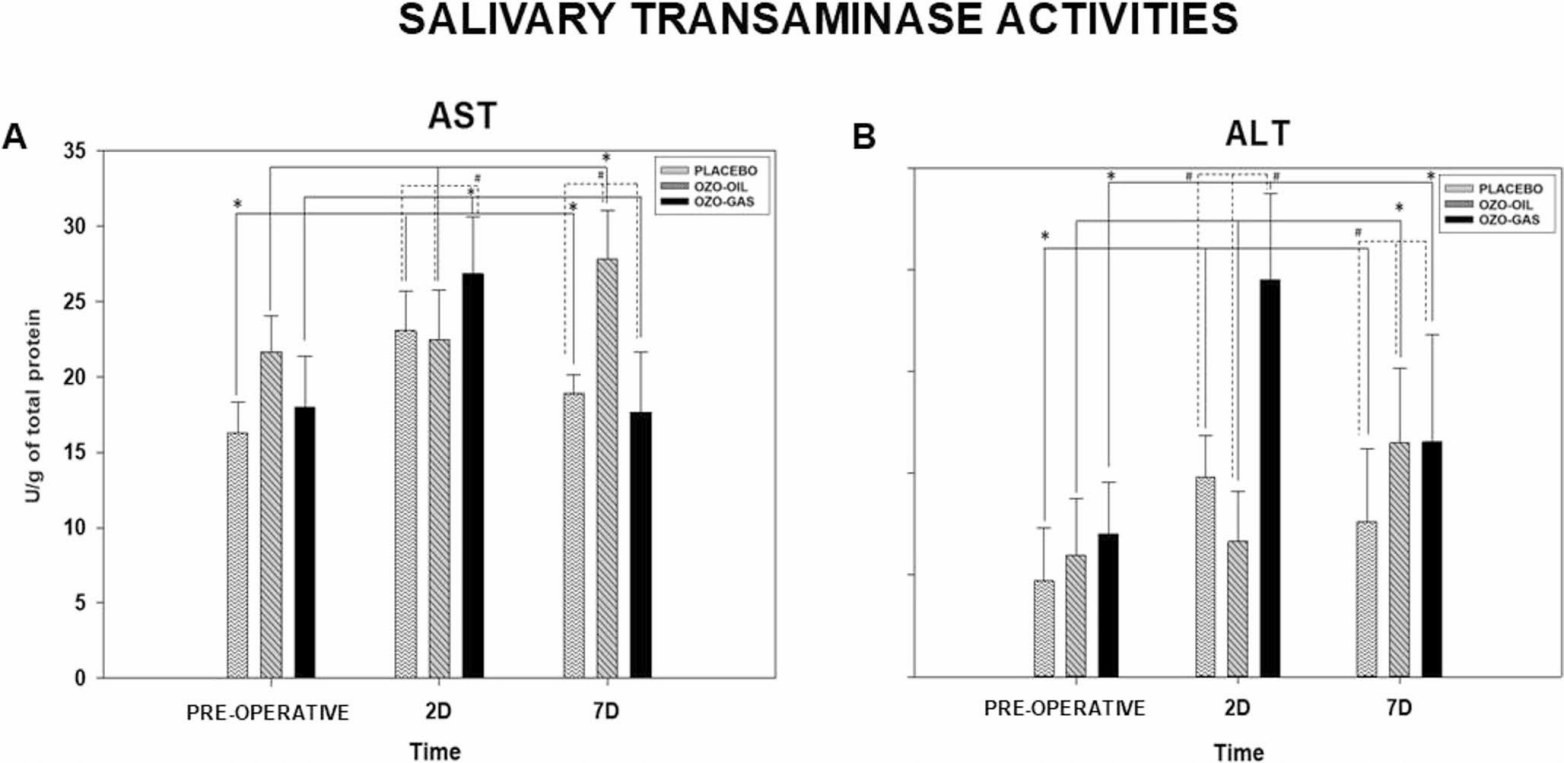
AST and ALT activities for the PLACEBO, OZO-OIL, and OZO-GAS groups, evaluated in the preoperative and postoperative (PO) periods. The symbol (*) indicates a statistical difference in the group × time comparison, while (#) denotes differences between groups within the same experimental period

AST activity showed significant temporal variation in all groups. In the PLACEBO group, AST increased on postoperative day 2 compared with baseline and decreased by day 7, with significant differences among time points (*P* < 0.05). In the OZO-OIL group, AST increased progressively, with day 7 values significantly higher than baseline and day 2 (*P* < 0.05). In the OZO-GAS group, AST rose on day 2 and returned to baseline levels by day 7, with significant differences between day 2 and the other periods (*P* < 0.05). No intergroup differences were observed at baseline. On postoperative day 2, AST activity was higher in OZO-GAS compared with PLACEBO and OZO-OIL, whereas on day 7, OZO-OIL showed higher AST activity than PLACEBO and OZO-GAS (Fig. 6).

ALT activity also showed significant temporal variation in all groups. In the PLACEBO group, ALT increased on postoperative day 2 compared with baseline and decreased by day 7, with significant differences among time points (*P* < 0.05). In the OZO-OIL group, ALT increased progressively, with day 7 values significantly higher than baseline and day 2 (*P* < 0.05). In the OZO-GAS group, ALT rose markedly on day 2 and declined on day 7, with significant differences among periods (*P* < 0.05). No intergroup differences were observed at baseline. On postoperative day 2, ALT activity differed among all groups (OZO-GAS > PLACEBO > OZO-OIL), and a similar pattern was observed on day 7 (OZO-GAS > OZO-OIL > PLACEBO) (Fig. 6).

#### Effects of ozone therapy on markers of salivary oxidative damage after tooth extraction

**Fig. 7:**
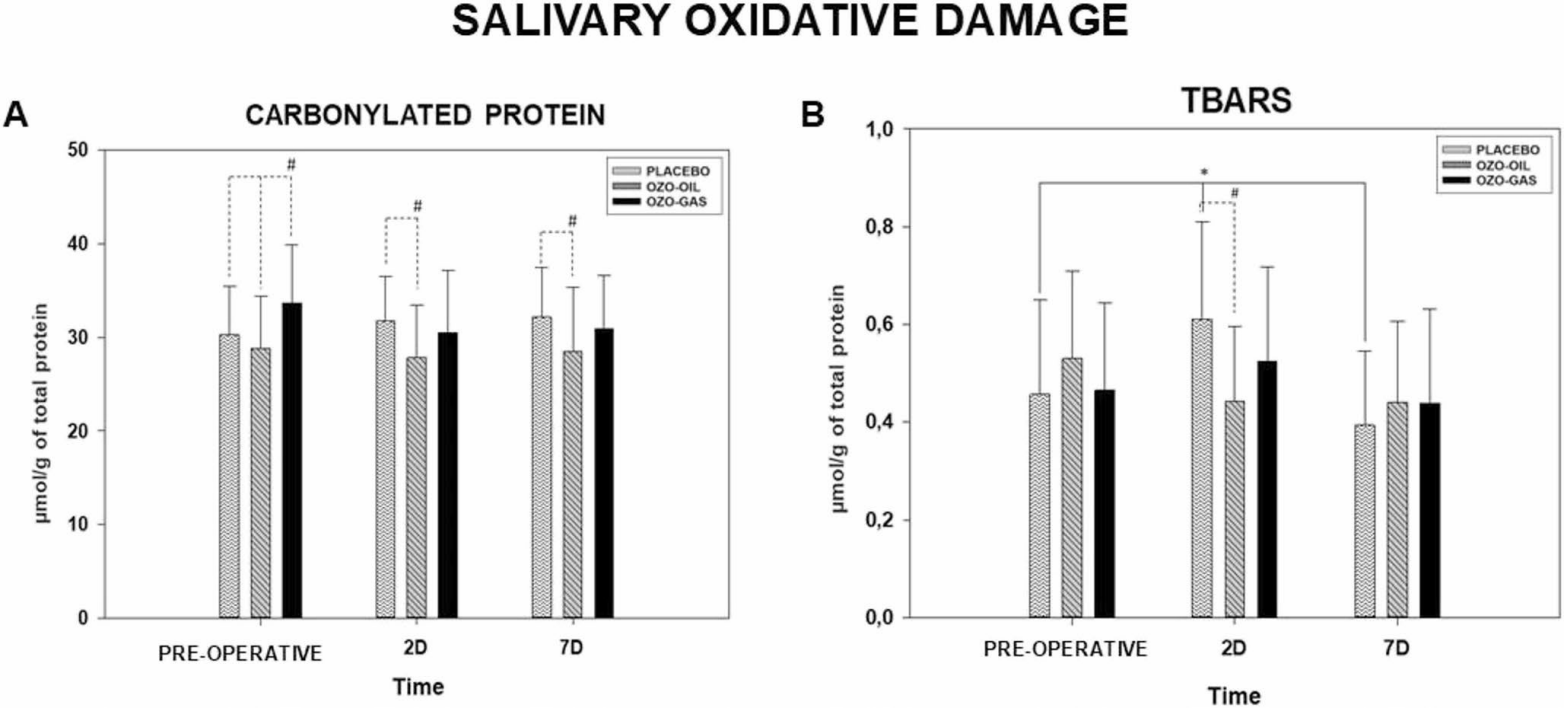
CP concentration and TBARS concentration for the PLACEBO, OZO-OIL, and OZO-GAS groups, evaluated in the preoperative and postoperative (PO) periods. For PC, the symbol (#) indicates differences between groups within the same experimental period. For TBARS, the symbol (*) represents a statistical difference in the group × time comparison, while (#) denotes differences between groups within the same experimental period

PC concentrations remained stable over time in all groups. Mean PC values ranged from 28.55 to 32.15 µmol/g of total protein across preoperative assessments and postoperative days 2 and 7, with no significant intragroup differences (*P* > 0.05). Intergroup analysis showed higher baseline PC levels in the OZO-GAS group compared with PLACEBO and OZO-OIL (*P* < 0.05). On postoperative days 2 and 7, the PLACEBO group exhibited higher PC concentrations than OZO-OIL, while both did not differ from OZO-GAS (Fig. 7).

TBARS concentrations showed limited temporal variation. In the PLACEBO group, TBARS increased on postoperative day 2 compared with baseline and decreased by day 7, with significant differences between day 2 and the other periods (*P* < 0.05). In contrast, OZO-OIL and OZO-GAS exhibited stable TBARS levels over time, with no significant intragroup changes (*P* > 0.05).No intergroup differences were observed at baseline or on postoperative day 7. On day 2, TBARS levels were higher in PLACEBO compared with OZO-OIL, while neither differed from OZO-GAS (Fig. 7).

#### Effects of ozone therapy on salivary non-enzymatic antioxidant defense after tooth extraction

**Fig. 8:**
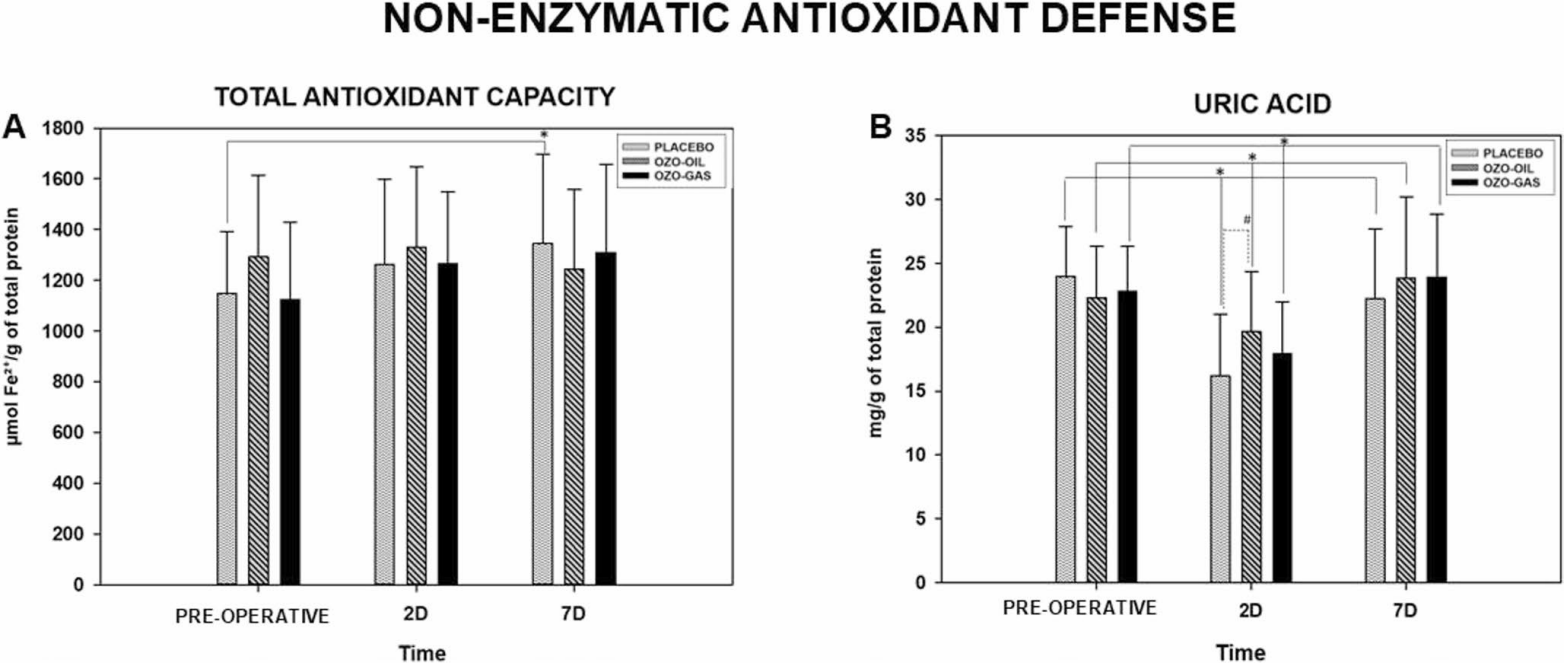
TAC activity and AU concentration for the PLACEBO, OZO-OIL, and OZO-GAS groups, evaluated in the preoperative and postoperative (PO) periods. For both parameters, the symbol (*) indicates a statistical difference in the group × time comparison, while for AU the symbol (#) denotes differences between groups within the same experimental period

Total antioxidant capacity showed minimal variation over time. In the PLACEBO group, values increased gradually, with a significant difference between baseline and postoperative day 7 (*P* < 0.05). In contrast, OZO-OIL and OZO-GAS exhibited stable antioxidant capacity with no significant intragroup changes (*P* > 0.05). No intergroup differences were observed among PLACEBO, OZO-OIL, and OZO-GAS at any time point (Fig. 8).

UA concentrations showed a transient postoperative reduction in all groups. Levels decreased on postoperative day 2 compared with baseline and returned to near-baseline values by day 7, with significant differences between day 2 and the other periods in all groups (*P* < 0.05) (Fig. 8).

## Discussion

The extraction of third molars is widely used as an experimental model to evaluate the effectiveness of drugs and adjuvant therapies in controlling postoperative (PO) symptoms such as pain, edema, and trismus, which justifies the study design adopted herein [3, 61, 62]. Ozone therapy, considered a relatively safe method with few reported complications and different modes of application, has emerged as a potentially useful non-pharmacological alternative in the postoperative period [63]. However, the lack of standardized protocols may limit its clinical effects.

In the present study, subperiosteal O₃ insufflation did not consistently modulate the evaluated outcomes, suggesting that this route of administration, under the analyzed conditions, may not be the most appropriate. Although O₃ exhibits bactericidal, anti-inflammatory, and analgesic properties described in the literature [16, 63], the clinical findings should be interpreted with caution. Pain assessment using the visual analogue scale (VAS) demonstrated better pain control in the ozone therapy groups compared with the PLACEBO group, particularly in the OZO-OIL group on postoperative day 2, which corresponds to the inflammatory peak. These results are consistent with Guinessi et al. [64], who reported accelerated soft-tissue healing and pain reduction following the use of ozonized oil.

The analysis of NAR partially corroborated the VAS findings, as both OZO-OIL and OZO-GAS prolonged the interval until the first rescue dose, with a longer interval observed in the OZO-OIL group. The OZO-GAS group exhibited lower total analgesic consumption compared with the PLACEBO group. Individual differences in pain perception, influenced by factors such as anxiety and stress, may have contributed to this variability [31].

Regarding edema, all groups showed a maximum increase on postoperative day 2, followed by a subsequent reduction, in agreement with previous studies [65, 66]. The OZO-GAS group presented significantly higher values at the edema peak. This finding may be interpreted in light of two main considerations: (i) limitations inherent to the manual method used for edema measurement, which has lower precision compared with more objective digital techniques such as photogrammetry or three-dimensional scanning [67, 68]. To minimize bias, all assessments were performed by a previously calibrated examiner following a standardized protocol; nevertheless, this limitation should be considered when interpreting the results; and (ii) tissue distension resulting from gas insufflation itself, which may contribute to greater local edema in the early postoperative period.

Maximum mouth opening followed the expected pattern, with worsening on postoperative day 2 and recovery by day 7 [3], with no statistically significant differences between groups, indicating that the evaluated therapies did not influence trismus under the tested conditions.

In an attempt to integrate clinical and biochemical findings, outcomes were correlated with salivary biomarkers. TP concentration did not vary between periods or groups, possibly due to standardization of surgical difficulty, procedures being performed by a single experienced surgeon, and control of surgical time [31, 69]. ACP and ALP, enzymes associated with bone metabolism and tissue injury, tend to increase after traumatic procedures and in contexts of greater inflammatory activity [32, 33, 70, 71]. Higher ACP levels were observed in the OZO-OIL group on postoperative day 7 compared with the OZO-GAS group, whereas ALP levels were higher in the OZO-GAS group on postoperative day 2, a finding consistent with the greater edema observed in this group. Discrepancies between ACP and ALP may reflect intrinsic limitations of saliva as a biosensor, as well as differences in analytical sensitivity, reinforcing the need to interpret these findings in conjunction with clinical parameters.

AST and alanine aminotransferase ALT, although classically used as markers of hepatic injury, are also present in saliva and are associated with inflammatory processes and cellular damage in oral conditions [32, 33, 72]. Higher AST levels were observed in the OZO-OIL group on postoperative day 7, as well as higher AST and ALT values in the OZO-GAS group on postoperative day 2, a pattern that parallels the findings of higher ALP activity and greater edema in this group. However, the low specificity of these markers requires caution in interpretation, and direct causal relationships cannot be established.

With respect to oxidative stress, uric acid (UA), the main non-enzymatic antioxidant in saliva responsible for approximately 70% of total antioxidant capacity, as well as total antioxidant capacity (TAC), TBARS, and protein carbonyls (PC) as indicators of oxidative damage, were evaluated [24, 28, 34, 73, 74]. Overall, these markers showed variations compatible with the expected acute inflammatory response following tooth extraction; however, these findings should be interpreted with caution, considering the exploratory nature of the analyses, the limited statistical power for biochemical endpoints, and potential baseline differences among groups. On postoperative day 2, the PLACEBO group showed increased levels of enzymes associated with cellular injury, such as ALP, AST, and ALT, concomitant with increased lipid oxidative damage and reduced UA levels,, a pattern consistent with the inflammatory peak described in the literature, although not allowing causal or definitive inferences regarding biochemical modulation. TAC did not show significant variations between periods, possibly reflecting its lower sensitivity to detect transient changes in the context of surgical trauma.

In contrast, during the same period, the OZO-OIL group exhibited a less pronounced reduction in UA, with significantly higher values compared with the PLACEBO group at the inflammatory peak, as well as lower levels of TBARS and PC, differences that may suggest a possible modulation of redox balance, but which should be considered preliminary and potentially influenced by baseline intergroup variations. In parallel, this group showed attenuation of the increase in tissue injury marker enzymes, whereas the OZO-GAS group did not demonstrate relevant modulation of antioxidant defense and exhibited higher enzyme activity, consistent with the clinical findings of greater edem**a**, however, these findings should be interpreted cautiously within the context of the study’s methodological limitations and exploratory design.

Regarding safety, adverse effects of ozone therapy are uncommon and, when present, are generally mild [75]. In this study, only one case of hypersensitivity was recorded in the OZO-GAS group, consistent with the low complication rate reported in larger samples. Nevertheless, ozone therapy does not replace steroidal and non-steroidal anti-inflammatory drugs, which remain first-line therapy for managing postoperative symptoms, particularly in preemptive protocols, despite their potential adverse effects [76].

In summary, within the limitations of this study, ozonized oil demonstrated favorable performance mainly in pain-related outcomes and in some markers associated with oxidative stress and inflammation. However, the biochemical findings should be interpreted with caution and regarded as exploratory, particularly due to the limited sample size, potential baseline intergroup differences, and the performance of multiple comparisons without formal correction, factors that restrict the generalizability of these results. In addition, the lack of patient blinding and the absence of a fully blinded placebo control may have influenced subjective outcomes, particularly pain perception. Moreover, the analysis of multiple biochemical markers, with a potential inflation of type I error, together with the short follow-up period, restricts the strength of causal inferences and precludes conclusions regarding sustained or long-term biochemical effects. Therefore, the results should be regarded as preliminary and underscore the need for standardized ozone therapy protocols (route, form of application, and dosage), as well as well-designed clinical trials with larger samples and extended follow-up to better clarify the clinical and biochemical effects of ozone therapy in minor oral surgery. Future studies are also needed to confirm the reproducibility and generalizability of biochemical findings across different clinical settings and populations.

## Conclusion

The findings indicate that local ozone therapy in oil dispersion may be considered a potential adjuvant approach for managing postoperative pain following mandibular third molar extraction. Although the findings related to pain control are supported by the clinical data, the observed changes in salivary biochemical parameters should be interpreted with caution due to the exploratory nature of the analyses and their methodological limitations. Therefore, these results should be regarded as preliminary and hypothesis-generating. Future studies with larger sample sizes and designs specifically planned to assess biochemical endpoints are needed to clarify their true biological significance and to determine whether these effects can be generalized beyond the surgical model and ozone therapy protocols investigated in this trial.

## Data Availability

The data that support the findings of this study are available from the corresponding author upon reasonable request.
